# An Unusual and Challenging Association of Renal Artery Aneurysm and Renal Cell Carcinoma That Underwent Laparoscopic Partial Nephrectomy

**DOI:** 10.7759/cureus.104070

**Published:** 2026-02-22

**Authors:** Akif Erbin, Ozgur D Tataroglu, Turan Ozdemir, Batu Akalin, Halil L Canat

**Affiliations:** 1 Department of Urology, Basaksehir Cam Sakura City Hospital, Istanbul, TUR

**Keywords:** aneurysm, carcinoma, kidney neoplasms, renal artery, renal cell

## Abstract

Renal artery aneurysms (RAAs) represent an uncommon condition, and their coexistence with renal cell carcinoma (RCC) is exceedingly rare. The surgical management of this combination is notably complex. In cases when the renal tumor is small in size and suitable for partial nephrectomy (PN), there are ongoing debates regarding the method of pedicle control during the procedure and the necessity of concurrent aneurysm repair. This case report study details a successful PN performed laparoscopically on a 58-year-old male patient with a saccular aneurysm measuring 11 x 10.65 mm that was identified involving the segmental artery at the site of branch formation in the distal of the right renal artery and a 23 x 24 mm cystic mass in the posterior to lower pole of the kidney, along with the treatment techniques associated with this condition in relation to current literature. In cases where the aneurysm is small, asymptomatic, and permits renal artery control, as demonstrated in our case, treatment may focus solely on the renal tumor.

## Introduction

The simultaneous presence of renal cell carcinoma (RCC) and renal artery aneurysm (RAA) presents an unforeseen and complex challenge for the surgical treatment of the patient. The simultaneous presence of RCC and RAA is very rare and has been reported in only a few cases [1]. Currently, RAAs, similar to renal tumors, are frequently detected incidentally during investigations for different reasons, attributed to the increasing use of imaging modalities. Based on the aneurysm's size and clinical presentation, different therapeutic strategies may be chosen, including monitoring or surgical intervention, such as aneurysmectomy and repair of the RAA [2].

In cases when the renal tumor is small in size and suitable for partial nephrectomy (PN), there are ongoing debates regarding the method of pedicle control during the procedure and the necessity of concurrent aneurysm repair [3]. This case report study details a successful PN performed laparoscopically on a patient with an 11 x 10.65 mm aneurysm at the renal artery bifurcation and a 23 x 24 mm cystic mass in the posterior to lower pole of the kidney, along with the treatment techniques associated with this condition in relation to current literature. Whereas simultaneous aneurysm repair and/or nephrectomy has been frequently discussed or performed in previously reported cases in the literature, the small (< 3 cm), asymptomatic nature of the aneurysm and its favorable anatomical location in our patient allowed surgical management to be directed exclusively toward tumor treatment, without necessitating aneurysm repair. 

## Case presentation

**Figure 1:**
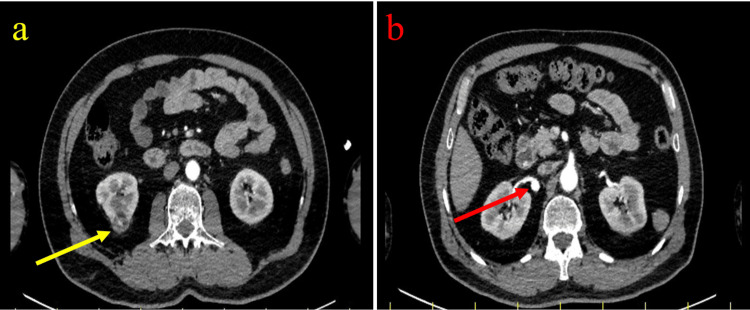
Axial contrast-enhanced CT images obtained in the arterial phase. (a) A 25-mm exophytic solid mass arising from the lower pole of the right kidney (yellow arrow). (b) A 1-cm saccular aneurysm located at the first bifurcation of the right renal artery (red arrow). Arterial-phase imaging allows clear delineation of both the renal mass and vascular anatomy, facilitating preoperative assessment and surgical planning.

**Figure 2:**
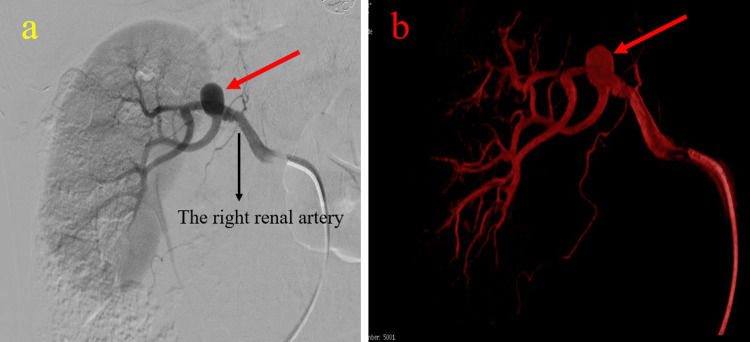
Selective renal artery digital subtraction angiography demonstrating a 1-cm saccular aneurysm located at the first bifurcation of the right renal artery (red arrows). (a) Conventional two-dimensional angiographic view outlining the aneurysm in relation to the main renal artery and its segmental branches. (b) Three-dimensional reconstructed angiographic image providing enhanced visualization of the aneurysm morphology and branching anatomy, aiding preoperative vascular assessment and surgical planning.

The required consent was obtained from the patient for the publication of this case report study. A 58-year-old male patient presented to our department with left lower back pain. The patient had hypertension and hypothyroidism, both managed with pharmacological intervention. No notable findings were observed during the physical examination. The basic laboratory tests were in normal ranges with a creatinine level of 0.82 mg/dL (normal range values: 0.7 to 1.3 mg/dL). In the patient's contrast-enhanced computed tomography (CT) and selective renal artery digital subtraction angiography examinations, a 25 mm exophytic lesion was seen in the lower pole of the right kidney (Figure 1a), and a 1 cm saccular aneurysm at the first bifurcation of the right renal artery was detected (Figures 1b, 2a, 2b). Nephrometric assessment of the renal mass demonstrated a R.E.N.A.L. score of 4, a PADUA score of 6, and a C-index of 3.8. The patient was discussed in the council, where cardiovascular surgeons were also present, and they stated that aneurysms smaller than 3 cm, asymptomatic, and not interfering with renal artery control should be managed conservatively with surveillance, while intraoperative repair should be reserved for cases in which the aneurysm involves the clamping site or demonstrates wall damage.

**Figure 3:**
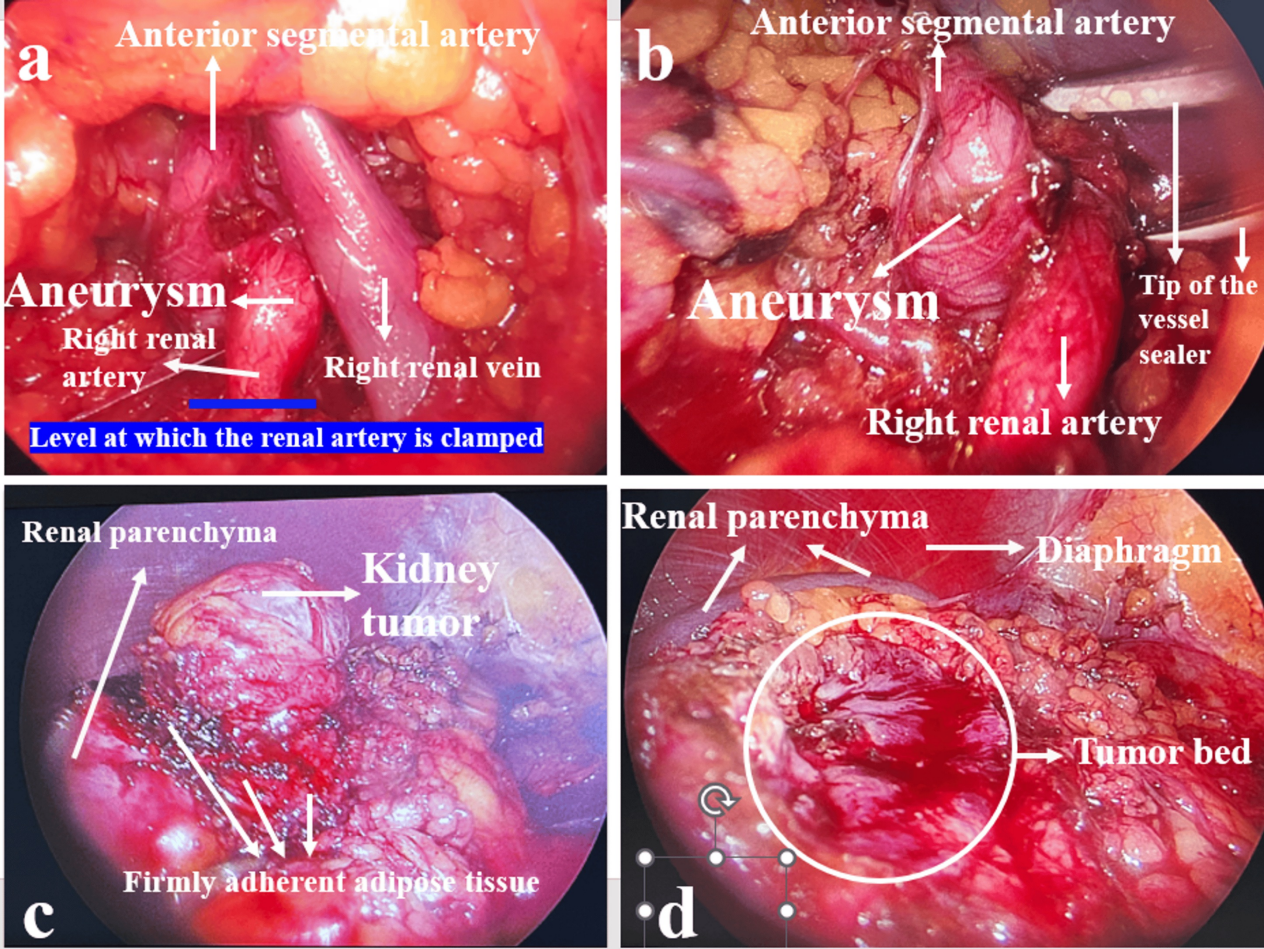
A renal artery aneurysm was detected at the branching point of the renal artery. The blue line indicates the level at which the renal artery is clamped with a bulldog clamp (a); the aneurysm was more clearly seen when the renal vein was retracted with an instrument (b); renal tumor with advanced reaction in perirenal fatty tissue (c); macroscopically clear tumor bed seen in renal parenchyma after tumor removal (d).

**Figure 4:**
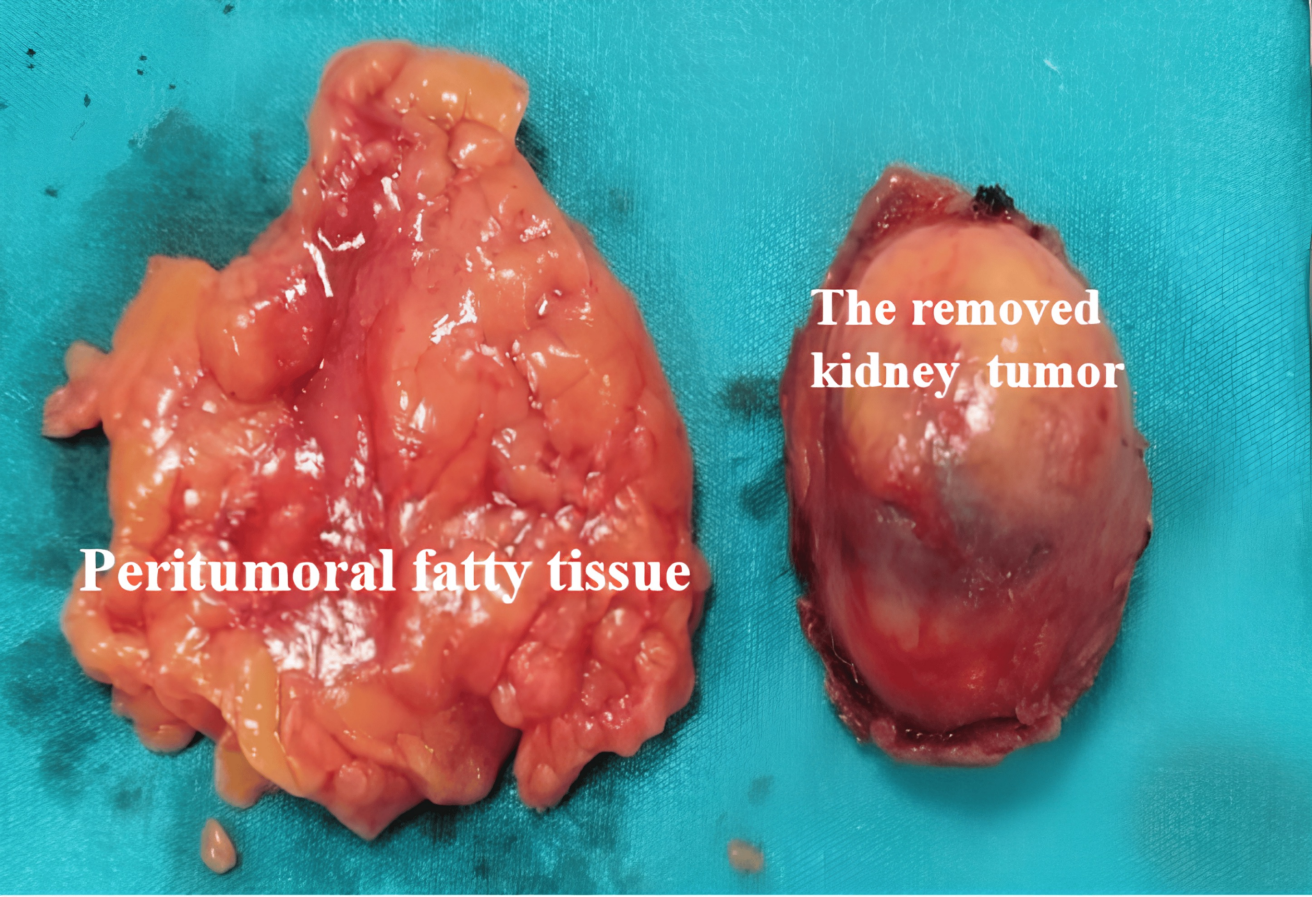
The tumor was removed together with the peritumoral fatty tissue without disrupting its integrity.

The laparoscopic PN was conducted without complications. The renal artery was released about 1.5 cm distal to the aneurysm, and it was noted that the aneurysm did not impact this region. The renal artery was clamped with a bulldog clamp at this level (Figures 3a, 3b). Adhesions between the mass and the surrounding peritumoral fatty tissue were significant, resulting in difficulty during the dissection of the tumor area (Figures 3c, 3d). The tumor was successfully removed, with a warm ischemia time of 24 minutes and an estimated blood loss of approximately 200 mL (Figure 4).

Following the removal of the urethral catheter on the first postoperative day, urine leakage from the daily drain was noted, necessitating the reinsertion of a urethral catheter. Nevertheless, a 6 Fr ureteral JJ stent was inserted into the patient on the third postoperative day due to urine leakage amounting to 400 cc. Notwithstanding this, urine leakage persisted for three weeks. As the drainage volume fell below 50 cc in the third postoperative week, the drain was withdrawn initially, followed by the removal of the urethral catheter three days later. The pathology report indicated clear-cell renal carcinoma, stage 1a, grade 1, with negative surgical margins.

## Discussion

Interpretation of the operative findings and postoperative course in this case highlights the feasibility of selective tumor-focused management, while the discussion of complications and comparison with previously reported cases emphasize the importance of individualized patient selection.

In patients with renal malignancies, RAAs are frequently identified incidentally during cross-sectional imaging, and most patients exhibit no symptoms related to RAAs. Various therapy options for RAAs include observation, surgical and endovascular repair, and radical or PN. However, there is significant debate about whether the patient should receive simultaneous aneurysm repair, the appropriateness of concurrent procedures when indicated, the management of cases where the aneurysm obstructs arterial clamping during PN, and the criteria for patients requiring radical nephrectomy. Current guidelines recommend follow-up for asymptomatic patients with an aneurysm of less than 3 cm in diameter. Aneurysms exceeding 3 cm in diameter, accompanied by symptoms such as flank pain, hematuria, or hypertension, are considered indications for elective surgical intervention [2]. As the present patient did not meet these criteria, a decision for surveillance was made by the cardiovascular surgery council.

The most suitable surgical intervention for RAAs is still controversial. The principal surgical procedures for an RAA encompass aneurysmectomy with primary closure or patch angioplasty, revascularization, and embolization. Aneurysm repair was considered unnecessary for our patient because of his asymptomatic condition, the small size of the aneurysm, and the fact that the RAA did not impede arterial clamping. If the RAA was located on the main renal artery in our case, as in other documented cases, its repair would likely be considered appropriate.

A comprehensive review identified only five documented cases of patients who underwent PN with concomitant ipsilateral RAA [4-8] (Table 1).

**Table 1:** Patients with concurrent RAA in cases undergoing PN Nr: not reported, PN: partial nephrectomy

Author	Age	Aneurysm	Mass	Pathology	Treatment
Foschi N et al., 2021 [4]	69	22 mm	9 cm	RCC/pT2a	open PN and RAA repair
Abreu AL et al., 2020 [5]	54 (median)	18 mm 30 mm	Nr	Angiomyolipoma RCC/ TNM; Nr	2 patients; robotic PN and RAA repair
Zani D et al., 2008 [6]	57	10 mm	25 mm	RCC/ pT1a	open PN and aneurysm repair
Subramonian K et al., 1998 [7]	49	25 mm	4 cm	RCC/ pT1a	open PN and RAA repair
Takamizawa A et al., 1989 [8]	63	18 mm	6 cm	RCC /pT1b	open PN and RAA repair

The most interesting cases of these cases were robotic partial PN and aneurysm repair in two patients [5]. Robotic partial nephrectomy was initially conducted with a concomitant ipsilateral kidney tumor, utilizing either the clamped approach (n = 1) or the unclamped zero-ischemia technique (n = 1). The RAA was accessed using robotic scissors, its interior was meticulously examined, and all clearly calcified regions were excised, maintaining sufficient non-calcified artery wall for rebuilding. Arterial reconstruction was performed using a continuous suture of 5-0 on a tapered needle. All other cases underwent conventional open PN and aneurysm repair. 

## Conclusions

No single management strategy exists for patients with synchronous renal artery aneurysm and renal malignancy, and treatment decisions should be individualized. Renal tumor resection with or without aneurysm repair may be performed using open, laparoscopic, or robotic approaches, depending on anatomical and clinical considerations. In selected cases where the aneurysm is small, asymptomatic, and does not interfere with renal artery control, as demonstrated in our case, surgical management may reasonably focus solely on the renal tumor. Nevertheless, given the inherent limitations of single-case evidence, this approach should be interpreted with caution and applied only in carefully selected patients with favorable anatomical and clinical characteristics.
